# Children with developmental coordination disorder exhibit altered cardiac autonomic control at rest and during exercise

**DOI:** 10.1016/j.clinsp.2025.100835

**Published:** 2025-11-21

**Authors:** Tatiane T.G. Draghi, Jorge L. Cavalcante Neto, Natália S. Valverde, Antonio R. Zamunér, Eloisa Tudella

**Affiliations:** a Centro Universitario Nossa Senhora do Patrocinio, Itu, Sao Paulo, Brazil; b Department of Human Sciences, Universidade do Estado da Bahia, Jacobina, Bahia, Brazil; c Departamento de Fisioterapia, Universidade Federal de São Carlos, São Carlos, Brazil; d Centro de Investigación en Neuropsicología y Neurociencias Cognitivas (CINPSI), Universidad Católica del Maule, Talca, Chile; e Laboratorio de Investigación Clínica en Kinesiología, Departamento de Kinesiología, Universidad Católica del Maule, Talca, Chile

**Keywords:** Motor skills disorder, Autonomic nervous system, Heart rate, Exercise, Physical fitness

## Abstract

**Objective:**

To analyze the cardiac autonomic control at rest, during, and after aerobic activity and assess the physical activity and performance of children with Developmental Coordination Disorder (DCD), and Typically Developing children (TD) in the 6-Minute Walk Test (6MWT).

**Methods:**

Forty-seven children participated in this case-control study, 21 with DCD. Cardiac autonomic control was assessed using heart rate variability using the Polar® V800 at rest in supine orthostatic postures, during the 6MWT, and during recovery seated in a chair. Physical activity was assessed using the Physical Activity Questionnaire for Children.

**Results:**

Children with DCD presented significant alterations in cardiac autonomic control during orthostatic (increased LFnu, *p* = 0.016; decreased HFnu, *p* = 0.016) and exercise (decreased Δ2LFnu, *p* = 0.002; and increased Δ2HFnu, *p* = 0.002) compared with children with TD. However, no significant differences in distance covered on the 6MWT or physical activity were observed.

**Conclusion:**

Children with DCD presented impaired cardiac autonomic control at rest and during the orthostatic rest stimulus before the 6MWT.

## Highlights

• Cardiac autonomic control in children with DCD is altered.
• Children with DCD exhibit higher sympathetic modulation compared to controls.
• Children with DCD exhibit reduced parasympathetic modulation compared to controls.
• This study reinforces the evidence of impaired autonomic control in children with DCD.

## Introduction

According to the Diagnostic and Statistical Manual of Mental Disorders, fifth edition, text revision (DSM-5 TR),^1^ Developmental Coordination Disorder (DCD) is a neurodevelopmental disorder characterized by deficits in the acquisition and execution of motor skills, which interfere with daily, academic, and leisure activities. The onset of these motor skills deficits is observed in early childhood and is not better explained by cerebral palsy, intellectual disability, muscular dystrophy, or any other known neurological condition.^1^ Children with Developmental Coordination Disorder (DCD) present a significantly decreased motor performance compared with age-matched children with Typical Development (TD).^1^^,^^2^ This motor deficit compromises the performance in sports, leisure activities, manual tasks, locomotion, and balance,^3^ decreasing quality of life.^4^ Furthermore, such aspects may potentially compromise physical fitness,^5^ restrict engagement in regular exercise practices, and influence Heart Rate (HR) control^6^^,^^7^ at rest and during exercise.

Sedentary behavior or low levels of exercise are associated with increased cardiovascular risk.^8^ In this sense, Heart Rate Variability (HRV) is a non-invasive tool that detects alterations in Cardiac Autonomic Control (CAC)^9^ and provides relevant prognostic findings for cardiovascular risk.^10^^,^^11^ Cavalcante Neto et al.^7^ investigated the CAC of children with DCD and TD in supine and orthostatic positions. It is important to note that, due to gravitational force, the upright posture imposes physiological stress on the cardiovascular system, requiring an appropriate autonomic response to compensate for the reduced venous return.^12^, ^13^, ^14^ Therefore, assessing the orthostatic stimulus provides deeper insights into autonomic nervous system function rather than relying solely on the supine position. Previous studies have shown that children with DCD present decreased motor performance and altered CAC. This cardiac autonomic dysfunction was characterized by increased sympathetic activity, limited autonomic adjustment to orthostatic stimuli, and decreased parasympathetic activity and complexity of CAC in the supine position.

On the other hand, many populations, including children and adolescents,^15^ benefit from regular exercise to improve cardiac autonomic control due to increased parasympathetic autonomic control and decreased sympathetic input.^15^, ^16^, ^17^ Furthermore, a previous study observed the reliability in assessing cardiorespiratory fitness using HRV during exercise by demonstrating that physically conditioned individuals presented more efficient autonomic adjustments during exercise than sedentary individuals.^18^ However, literature lacks studies examining the autonomic nervous system modulation during exercise in children with DCD.

In this context, considering the differences in CAC between children with DCD and TD in supine and orthostatic resting positions, it is relevant to investigate HRV in other moments, such as during exercise. Assessing CAC during exercise may also improve knowledge regarding this mechanism, facilitate the planning of intervention programs according to individual needs, improve functionality, and increase the participation of children with DCD in society. Therefore, this study aimed to analyze CAC at rest, during, and after exercise and to assess the physical activity and performance of children with DCD and TD in the 6-Minute Walk Test (6MWT).

## Methods

It was a case-control study in accordance with the STROBE statement.^19^ Children of both sexes aged between seven and ten years were divided into two groups: children with DCD (DCDG) and Typical Development (TDG). All participants were assessed according to the four criteria of the Diagnostic and Statistical Manual of Mental Disorders - Fifth edition, text revision (DSM-5:)^1^ A (significantly lower motor performance in motor performance assessment), B (interference of low motor performance in daily life, school, and leisure activities), C (motor impairments present since the early stages of child development), and D (motor condition not associated with intellectual or visual impairment or neurological conditions). Children meeting all four criteria were allocated to the DCDG, whereas those in the TDG presented no motor performance difficulties according to the Movement Assessment Battery for Children – Second Edition (MABC-2). Children with orthopedic, cardiac, or other clinical conditions compromising the evaluation (e.g., flu, antibiotics, anti-inflammatory, or antiallergy medication) were excluded.

Children were recruited from a private school in São Carlos (São Paulo, Brazil). This study was approved by the research ethics committee of the Federal University of São Carlos (UFSCar) (no. 3.298.299 and 89,993,118.8.0000.5504), following the Declaration of Helsinki and resolution n° 466 National Health Council.

**Fig. 1:**
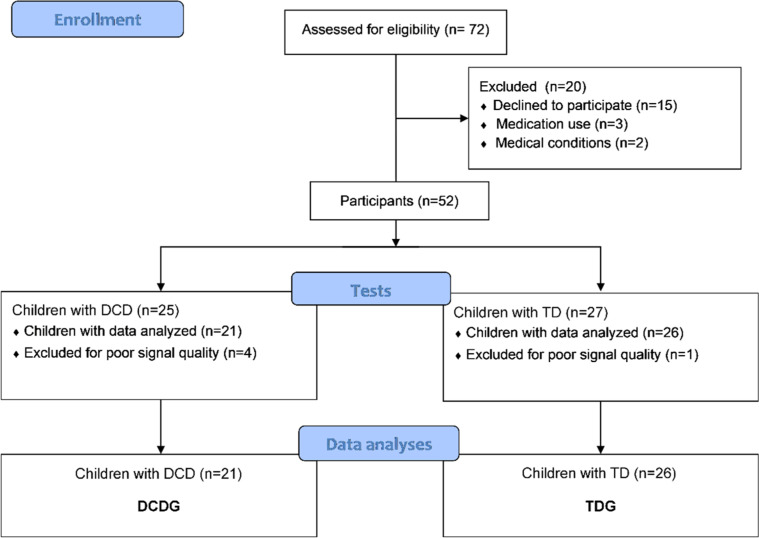
Flowchart of recruitment and participant selection.

First, 158 children were invited through an invitation letter sent to their parents. Seventy-two children met the preliminary criteria and were invited to participate and visit the laboratory at the university for data collection. Of these, 52 children agreed to participate in the second stage. Parents or guardians signed an informed consent form, whereas children signed the informed assent form before participating. Five children were excluded due to interference or artifacts on collected data; therefore, 47 children completed all stages of the study (Fig. 1). A power analysis was conducted considering the mean values of the significant delta changes of Heart Rate Variability (ΔHRV) indices Low Frequency (LF) and High Frequency (HF) in normalized units (nu), and LF/HF ratio, using the OpenEpi platform (https://www.openepi.com/Power/PowerMean.htm). The sample of 47 children had a power of 93.61 %. The authors used the proportion of 1.2 controls per case.

### Experimental protocol

The first stage of the study was conducted in the school, while the second was carried out in the Development and Function Evaluation Laboratory (LADeF) of the Department of Physical Therapy at UFSCar. Guardians received an informed consent form, the Developmental Coordination Disorder Questionnaire, and a demographic questionnaire during the first stage. According to the responses, those children who met the inclusion criteria were selected for motor performance assessment using the MABC-2, followed by an evaluation appointment at LADeF.

**Fig. 2:**
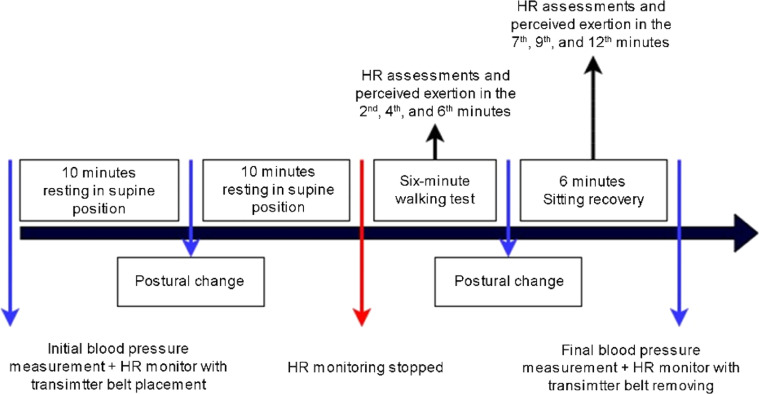
Illustration of the experimental protocol.

Anamnesis and anthropometric data were collected during the second stage. Right after, children received information about HR and blood pressure at rest and during exercise, familiarized with the environment and resting positions (Fig. 2), and answered the Physical Activity Questionnaire for Children (PAQ-C) assisted by their guardians.

### Assessment instrument

#### Developmental coordination disorder questionnaire

Guardians answered the Developmental Coordination Disorder Questionnaire to assess criterion B of the DSM-5. This questionnaire has 15-items^20^ with scores based on a Likert scale ranging from 1 (not at all like your child) to 5 (extremely like your child). Scores were interpreted according to the age range.

#### Movement assessment battery for children

Motor performance (i.e., criterion A of the DSM-5) was assessed using the MABC-2^21^ age band two, which assesses three components: manual dexterity, aiming and catching, and balance during approximately 20-minutes.^21^ The total standard score estimates the overall motor performance and is calculated as the sum of the standard scores. Scores below 67 indicated children with low motor performance (i.e., children with DCD), and scores above this threshold indicated children without motor performance difficulties (i.e., typically developing).^21^ The MABC-2 tests were administered by two qualified pediatric physiotherapists with approximately five years of experience in this assessment.

#### Demographic factors

Sociodemographic information and the health and developmental history of children were collected using an in-house developed questionnaire. The questionnaire consisted of 32 objective questions and evaluated aspects regarding sex, age range, motor development history from birth to the current age, possible neurological alterations, known diagnoses of autism spectrum disorder, attention deficit hyperactivity disorder, or other disorders answered by the guardians. This information was essential to fulfill criteria C and D of DSM-5.^1^

#### Anthropometric measurements

The weight and height of children were assessed using a WISO® W602 digital scale and WCS® Wood portable stadiometer, respectively. Body mass index was calculated, and waist circumference was measured using an extendable measuring tape (Sanny®).

#### Physical activity questionnaire for children

Physical activity level was assessed using the PAQ-C, translated and adapted to the Brazilian culture,^22^ and used in children with DCD.^7^ This questionnaire consisted of nine questions about participation in sports and games, exercise at school, and during leisure time (including weekends). PAQ-C assesses the physical activity of children over the last seven days, and each question is scored from 1 (very sedentary) to 5 (very active). Children were categorized as sedentary (scores below 3) or active (scores above 3).

#### Cardiac autonomic control

The cardiac autonomic control was assessed by calculating the HRV using R-R intervals from an electrocardiogram, using a HR monitor (Polar® V800) and a chest strap (Polar® H10). The HRV analysis was conducted using linear methods in the time domain, including the indexes: standard deviation of the normal-to-normal intervals and root mean square of the differences between adjacent RR intervals (in milliseconds). The frequency domain was assessed using autoregressive analysis, which quantified the Low Frequency (LF) (0.04 to 0.15 Hz) and High Frequency (HF) (0.15 to 0.4 Hz) in absolute units. Normalized LF or HF (nu) were obtained by calculating the ratio between these parameters and the total variance of RR intervals, subtracted from the very low frequency (0.003 to 0.04 Hz), and multiplied by 100.^23^

RR intervals were recorded at four different moments: resting in the supine position for 10 min (M1); during orthostatic posture for 10 min (M2); during the 6MWT (M3); and seated in a chair, with feet supported, for the following six minutes after the test (recovery period; M4). Participants maintained spontaneous breathing throughout the procedure, and respiratory rate was monitored during resting conditions and recovery, via visual inspection of thoracoabdominal movements. No participant exhibited a respiratory rate below 9 bpm or above 20 bpm during the assessment periods. HRV analysis considered 256 consecutive beats at each moment and was performed using Kubios HRV Standard software, version 3.3.1 (Kubios Oy, Kuopio, Finland).

#### Six-minute walk test

The 6MWT is an easy-to-apply test that simulates aerobic activity and consists of walking in a 30-meter corridor (regular and free of obstacles) for six minutes.^24^ Children were instructed to walk at the fastest pace possible without running to complete the maximum number of laps.^24^ Before starting, children received instructions about the test (“during the six minutes, you should walk as fast as you can, without running”), while standardized stimuli (“you are doing great, five minutes to go”) were provided every minute during the test. At the end of the six-minute period, children stopped and sat comfortably in a chair with their feet supported for another six minutes, to analyze the recovery period. RR intervals were continuously recorded during the entire 6MWT (M3) and in the recovery period (M4).

#### Children’s OMNI-walk/run scale of perceived exertion

The Children’s OMNI-walk/run Scale of Perceived Exertion^25^ was used to guide the progression of the 6MWT, monitor the perceived exertion, and provide immediate feedback for adjustments during the test. This scale includes illustrations of a child climbing a ramp, the corresponding score, and descriptions of fatigue to improve the visualization and comprehension by children. A score of 0 corresponded to “not tired at all”, while a score of 10 denoted “very, very tired”.

### Statistical analysis

Data normality and variance homogeneity were assessed using the Shapiro-Wilk test and Levene test, respectively. Characterization data and variables of interest between groups at M1, M2, M3, and M4 were compared using an unpaired Student’s *t*-test or Mann-Whitney’s *U test*. These tests were also used to analyze changes (Δ) from M1 to M2 (Δ1, calculated as M2 values minus M1 values), M2 to M3 (Δ2, calculated as M3 values minus M2 values), and M3 to M4 (Δ3, calculated as M4 values minus M3 values). Deltas were calculated for each participant. All analyses were conducted using the Statistical Package for the Social Sciences® version 25.0. The significance level was set at 5 %.

## Results

Age, anthropometric data, and physical activity were not significantly different between the DCDG and TDG. Children in the TDG presented higher scores in MABC-2, as expected, than those in the DCDG (*p* < 0.001). Table 1 presents the characteristics of the included children.

**Table 1:** Characteristics of participants in the DCDG and TDG groups.

Variables	DCDG (*n* = 21)	TDG (*n* = 26)	p-value
Age (years) a	8 (7;9)	8 (7; 9)	0.89
Sex (Male/Female) b	(11/10)	(13/13)	0.87
TSS MABC-2 (score) c	55.76 ± 6.73	86.03 ± 9.32	<0.001^d^
Weight (kg) c	34.79 ± 8.95	37.85 ± 11.31	0.31
Height (cm) c	135 ± 7	137 ± 9	0.29
PAQ-C (score) c	1.38 ± 0.49	1.57 ± 0.50	0.18
Waist circumference (cm) a	66 (61;75.50)	69.50 (59.12;80.25)	0.89
BMI (kg/m^2^) a	17.63 (15.31;20.94)	19.38 (15.92; 22.32)	0.64

DCDG, Children with DCD group; TDG, Children with typical development group; TSS MABC-2, Total test Score of the Movement Assessment Battery for Children; PAQ-C, Physical Activity Questionnaire for Children; BMI, Body Mass Index; cm, centimeter; kg, kilograms; m, meters.

a Non-parametric data presented as median, first and third quartile.

b Chi-Square test.

c Parametric data presented as mean and standard deviation.

d *p* < 0.05 Student’s *t*-test.

### Heart rate variability and aerobic capacity

The HRV indices from both groups are presented in Table 2. No significant differences were observed between groups regarding cardiac autonomic control in supine (M1) posture (*p* > 0.05). On the other hand, the DCDG presented greater LFnu (*p* = 0.01) and lower HFnu (*p* = 0.01) during standing (M2), compared to the TDG, thus suggesting higher sympathetic and attenuated parasympathetic modulation response to the orthostatic stimulus in children with DCD. Additionally, as observed in Table 3, the autonomic adjustment to exercise was decreased in the DCDG, evidenced by reduced Δ2LFnu and Δ2HFnu (*p* = 0.002). No significant differences were observed for other deltas, nor regarding the percentage of maximum HR achieved and distance covered during 6MWT (percentage of predicted).

**Table 2:** Comparison of heart rate variability indices between groups.

Variables	Moments	Mean and standard deviation		p-value
		DCDG (*n* = 21)	TDG (*n* = 26)	
Mean iRR (ms)^a^	Supine	633.2 ± 144.9	673.1 ± 68.3	0.28
	Standing	568.6 ± 43.6	576.9 ± 59.7	0.70
	6MWT	470.8 ± 57.3	470.3 ± 46.4	0.48
	Recovery	604.4 ± 55.0	616.5 ± 70.3	0.51
LF (ms^2^)	Supine	797.6 ± 536.1	956.4 ± 1017.3	0.52
	Standing	610.8 ± 100.5	563.0 ± 152.1	0.19
	6MWT	104.0 ± 21.6	67.3 ± 10.5	0.32
	Recovery	766.9 ± 787.5	867.3 ± 852.9	0.67
HF (ms^2^)	Supine	1419.2 ± 2223.2	1193.3 ± 1017.3	0.52
	Standing	310.9 ± 102.3	351.9 ± 89.6	0.79
	6MWT	28.6 ± 8.6	14.8 ± 3.6	0.18
	Recovery	513.7 ± 427.2	642.8 ± 858.0	0.50
LF (nu)^a^	Supine	48.9 ± 19.9	48.6 ± 15.1	0.95
	Standing	71.6 ± 12.0	61.8 ± 13.8	0.01^b^
	6MWT	79.6 ± 10.9	83.5 ± 9.6	0.19
	Recovery	58.3 ± 12.8	53.4 ± 22.4	0.67
HF (nu)^a^	Supine	50.8 ± 19.9	50.2 ± 14.8	0.91
	Standing	28.4 ± 12.0	38.2 ± 13.8	0.01*
	6MWT	20.4 ± 10.9	16.5 ± 9.6	0.19
	Recovery	41.5 ± 12.7	44.7 ± 18.8	0.49
LF/HF	Supine	1.46 ± 1.47	1.16 ± 0.83	0.05
	Standing	3.90 ± 3.79	2.73 ± 2.43	0.38
	6MWT	6.2 ± 6.1	8.8 ± 11.6	0.19
	Recovery	1.68 ± 0.97	3.78 ± 6.79	0.13

DCDG, Children with DCD group; TDG, Children with typical development group; HRV, Heart Rate Variability; iRR, RR interval; LF, Low Frequency; HF, High Frequency; ms, milliseconds; nu, normalized units; 6MWT, 6-Minute Walk Test.

a Pparametric data.

b *p* < 0.05 Student’s *t*-test.

**Table 3:** Comparison of delta changes in the heart rate variability indices (∆HRV) and 6MWT variables between groups.

Variables	Moments	Mean and standard deviation		p-value
		DCDG	TDG	
∆HRV indices				
Mean (iRR)^a^	∆1	−36.5 ± 196.4	−104.0 ± 46.5	0.15
	∆2	−97.8 ± 64.3	−106.6 ± 56.5	0.84
	∆3	131.7 ± 38.9	135.8 ± 45.0	0.73
LF (ms^2^)	∆1	−212.3 ± 637.0	−413.3 ± 1047.7	0.44
	∆2	−506.8 ± 458.0	−495.8 ± 734.6	0.25
	∆3	662.2 ± 781.9	791.1 ± 835.3	0.58
HF (ms^2^)	∆1	−1131.5 ± 2126.3	−897.2 ± 1375.5	0.68
	∆2	−282.3 ± 474.3	−337.1 ± 435.1	0.48
	∆3	483.6 ± 422.0	625.6 ± 846.1	0.46
LF (nu)^a^	∆1	23.5 ± 29.6	14.3 ± 24.9	0.29
	∆2	8.0 ± 14.1	21.7 ± 12.4	0.002*
	∆3	−19.3 ± 21.4	−29.6 ± 22.0	0.11
HF (nu)^a^	∆1	−18.7 ± 26.2	−13.3 ± 24.6	0.49
	∆2	−8.0 ± 14.1	−21.7 ± 12.4	0.002^b^
	∆3	19.6 ± 19.7	27.7 ± 17.9	0.14
LF/HF	∆1	2.85 ± 4.21	1.77 ± 2.37	0.18
	∆2	3.36 ± 4.95	5.76 ± 9.88	0.55
	∆3	−5.34 ± 5.57	−4.62 ± 12.93	0.80
6MWT variables				
Distance (m)^a^		450.3 ± 61.7	441.2 ± 53.7	0.60
% predicted^a^		83 ± 9.2	81 ± 9.99	0.66
% HRmax		63 (59; 68)	66.5 (58; 71)	0.49

DCDG, Children with DCD group; TDG, Children with typical development group; HRV, Heart Rate Variability; iRR, RR Interval; LF, Low Frequency; HF, High Frequency; ms, milliseconds; nu, normalized units; 6MWT, 6-Minute Walk Test; ∆1, Changes between orthostatic and supine rest position; ∆2, Changes between exercise and orthostatic rest position; ∆3, Changes between recovery and exercise; total distance in 6MWT; m, meters; % predicted, percentage of predicted distance ( % predicted = 145.343 + [11.78 × age (years)] + [292.22 × Height (m)] + [0.611 × difference in HR (bpm)] – [2.684 × Weight (kg)]); % HRmax, Percentage of maximum heart rate.

a Parametric data presented as mean and standard deviation.

b *p* < 0.05 Student’s *t*-test.

## Discussion

This study demonstrated that children with DCD were characterized by decreased parasympathetic and increased sympathetic modulation during orthostatic stimulus, and a decreased autonomic adjustment to exercise compared with children with TD. These findings are in accordance with Chen et al.,^26^ who showed a higher LF/HF index in children with DCD than in children with TD during awake orthostatic posture. Although HRV was assessed during cognitive tasks, these observations corroborate the present findings regarding cardiac autonomic imbalance in children with DCD.

The blunted autonomic control adjustment presented by the DCDG during the 6MWT, characterized by a lower increase in LFnu (i.e., sympathetic modulation) and a lower decrease in HFnu (i.e., parasympathetic modulation), must be highlighted. These findings are consistent with the study of Cavalcante Neto et al.,^7^ which demonstrated that children with DCD exhibited limited autonomic adjustment during the transition from supine to orthostatic position compared with children with TD. In this sense, CAC responses may be limited in children with DCD during either the transition between supine and orthostatic position or the transition between orthostatic position and exercise. Moreover, since the DCD group presented a greater sympathetic activity already during standing, the authors may infer that a ceiling effect might have occurred during exercise, not allowing the sympathetic modulation to increase further.

Previous studies have shown that aerobic capacity was lower in children with DCD than in children with TD.^27^^,^^28^ The autonomic nervous system continuously modulates several hemodynamic and vascular responses during exercise (e.g., HR, cardiac contractility, and systemic adjustments), such as increased stroke volume and cardiac output^29^ to supply the metabolic demands of active muscles, including oxygenation and washout of metabolic end-products.^30^ In this sense, a limited autonomic adjustment characterized by lower sympathetic input and lesser vagal withdrawal could contribute to the early sensation of fatigue and lower aerobic capacity.

On the other hand, a decreased autonomic adjustment to exercise is present in individuals with low physical activity levels.^18^ The findings of this study also indicated no significant differences in physical activity and performance in the 6MWT between children with DCD and TD. Both groups covered <100 % of the predicted distance and scored less than two in the PAQ-C, indicating a very sedentary profile.^22^ This absence of differences between groups reinforces that cardiac autonomic alterations may be independent of physical activity and associated with the pathophysiological process of the DCD condition. Moreover, considering the lack of significant differences in aerobic functional capacity, cardiac autonomic alteration may precede changes associated with low aerobic capacity. Therefore, further studies are needed to elucidate whether the impaired autonomic nervous system control is a factor contributing to low physical activity in children with DCD or a simple negative adaptation inherent to this population.

Children in this study presented inadequate physical activity levels regardless of their motor condition. Physical activity tends to decrease after the age of five,^31^ and these findings raise an important public health alert since physical inactivity is one of the leading causes of premature mortality and increases public health expenses.^32^ Also, lack of exercise leads to sedentary behavior, which is directly associated with decreased HRV and a predictor of cardiovascular diseases.

HR and the predicted maximum HR during exercise were not significantly different between groups, indicating that children from both groups reached a similar exercise intensity during the 6MWT. Importantly, the predicted maximum HR during this test was close to 62 %, a level considered submaximal.^33^

Although the number of children enrolled may be considered a limitation of the study, the populations studied presented significant differences in the responses to orthostatic resting position and adaptation to exercise. Another possible limitation was the type of exercise. The 6MWT is a safe and valid test to assess aerobic capacity, but it may not have evoked sufficient exercise intensity, as demonstrated by the HR values of both groups. Thus, possible differences in the cardiac autonomic control of children with DCD could not be detected. Lastly, daily physical activity levels were not assessed in this study, which could clarify possible differences in the daily physical activity of children. Nevertheless, a recent study that monitored physical activity using accelerometers identified the prevalence of sedentary behavior in children at risk for DCD and children with TD.^34^

Future studies with a larger number of children and different types of exercise could provide additional findings. Also, the HRV may properly and safely assist intervention programs and help with the assessment of physiological improvements in children with DCD, mainly regarding the cardiovascular condition, exercise capacity, and cardiovascular risk.

In conclusion, children with DCD presented increased sympathetic and decreased parasympathetic autonomic modulation during the orthostatic rest position. Limited autonomic adjustment during exercise, characterized by less intense sympathetic input and less intense vagal withdrawal, was also found regardless of the physical activity and aerobic functional capacity. These findings strengthen the need to consider cardiovascular modulation mechanisms during the assessment and intervention of children with DCD.

## CRediT authorship contribution statement

**Tatiane T.G. Draghi:** Conceptualization, Methodology, Data curation, Investigation, Formal analysis, Writing – original draft. **Jorge L. Cavalcante Neto:** Conceptualization, Methodology, Investigation, Formal analysis, Writing – original draft. **Natália S. Valverde:** Methodology, Investigation, Formal analysis, Writing – original draft. **Antonio R. Zamunér:** Conceptualization, Methodology, Formal analysis, Writing – review & editing. **Eloisa Tudella:** Conceptualization, Methodology, Formal analysis, Writing – review & editing.

## Declaration of competing interest

The authors declare no conflicts of interest.

## Data Availability

The datasets generated and/or analyzed during the current study are available from the corresponding author upon reasonable request.

## Appendix Supplementary materials

Image, application 1

Image, application 2
