# Supplementary material for: Children with developmental coordination disorder exhibit altered cardiac autonomic control at rest and during exercise
Source: Clinics (Sao Paulo). 2025 Nov 21;80:100835. doi: 10.1016/j.clinsp.2025.100835 (PMC12681521; doi:10.1016/j.clinsp.2025.100835)
Supplement: Supplementary file 2 [file mmc2.docx]

Article Title: Children with developmental coordination disorder exhibit altered cardiac autonomic control at rest and during exercise
Corresponding Author: Dr. Antonio Roberto Zamunér


. All manuscripts must conform to specific study guidelines. Examples: Clinical Trials should follow the CONSORT Statement rules. Observational Studies (Cohort, case-control, and cross-sectional studies) should follow the STROBE Statement. Systematic Reviews and Meta-Analysis should follow the PRISMA guidelines. Diagnostic and Prognostic Studies should follow the STARD guidelines. Animal and Clinical Study should follow the ARRIVE guidelines. Please mention this in the text.

This study followed the STROBE Statement guidelines for observational studies and this information was included in the manuscript as well as the STROBE checklist was uploaded as supplementary file.

. Highlights are 3 to 5 bullet points that help increase the discoverability of your article through search engines. These bullet points are meant to be short (85 CHARACTERS OR FEWER, INCLUDING SPACES) and should summarize the content of your research. Please make sure to include terms that you know your readers will be looking for online. Please have a look at the examples: <https://www.elsevier.com/researcher/author/tools-and-resources/highlights>.

This information is now provided in the Highlights file.

. Please include the ORCID iD of all authors of the manuscript in the main document file.

This information was provided in the “Essential title page information” file

. Author contribution: CRediT statements should be provided - <https://www.elsevier.com/researcher/author/policies-and-guidelines/credit-author-statement>

This information was provided in the “Essential title page information” file
